# Pacemaker Activity of the Human Sinoatrial Node: An Update on the Effects of Mutations in *HCN4* on the Hyperpolarization-Activated Current

**DOI:** 10.3390/ijms16023071

**Published:** 2015-01-29

**Authors:** Arie O. Verkerk, Ronald Wilders

**Affiliations:** Department of Anatomy, Embryology and Physiology, Academic Medical Center, University of Amsterdam, 1105 AZ Amsterdam, The Netherlands; E-Mail: a.o.verkerk@amc.uva.nl

**Keywords:** sinoatrial node, pacemaker activity, funny current, hyperpolarization-activated current, HCN4, ion channelopathies, action potential clamp, computer simulation

## Abstract

Since 2003, several loss-of-function mutations in the *HCN4* gene, which encodes the HCN4 protein, have been associated with sinus node dysfunction. In human sinoatrial node (SAN), HCN4 is the most abundant of the four isoforms of the HCN family. Tetramers of HCN subunits constitute the ion channels that conduct the hyperpolarization-activated “funny” current (*I*_f_), which plays an important modulating role in SAN pacemaker activity. Voltage-clamp experiments on HCN4 channels expressed in COS-7, CHO and HEK-293 cells, as well as in *Xenopus* oocytes have revealed changes in the expression and kinetics of mutant channels, but the extent to which especially the kinetic changes would affect *I*_f_ flowing during a human SAN action potential often remains unresolved. In our contribution to the Topical Collection on Human Single Nucleotide Polymorphisms and Disease Diagnostics, we provide an updated review of the mutation-induced changes in the expression and kinetics of HCN4 channels and provide an overview of their effects on* I*_f_ during the time course of a human SAN action potential, as assessed in simulated action potential clamp experiments. Future research may solve apparent inconsistencies between data from clinical studies and data from *in vitro* and *in silico* experiments.

## 1. *HCN4* and Familial Sick Sinus Syndrome

The “sick sinus syndrome” has been defined as the “intrinsic inadequacy of the sinoatrial node (SAN) to perform its pacemaking function due to a disorder of automaticity and/or inability to transmit its impulse to the rest of the atrium” [1]. In 2003, Schulze-Bahr *et al.* [2] were the first to link familial sick sinus syndrome to mutations in the hyperpolarization-activated cyclic nucleotide-gated (HCN) gene family that mediates the hyperpolarization-activated “pacemaker current” or “funny current” (*I*_f_) in the heart (for reviews, see [3,4,5,6,7]). The HCN channel family comprises four members, HCN1–HCN4, which can form HCN channels in homomeric, as well as heteromeric tetramers. The four HCN family members display distinct expression patterns in the body (for reviews, see [4,8,9,10]), but the dominant HCN transcript in the human SAN is HCN4 [11]. It is, therefore, not surprising that the *HCN4* locus has been identified as a modulator of heart rate in a genome-wide association study (GWAS) [12] and that reduced *HCN4* expression due to endurance exercise is associated with a lower resting heart rate [13]. A reduced *HCN4* expression is also associated with a lower heart rate in animal models of heart failure [14] and aging [15]. Less obviously, the *HCN4* locus had already been identified as a susceptibility locus for atrial fibrillation (AF) in another GWAS [16].

Both HCN1-deficient [17] and HCN2-deficient [18] transgenic mice may display a sick sinus syndrome phenotype. However, till now, reports of mutations affecting *I*_f_ in the human heart have been restricted to the *HCN4* gene [2,19,20,21,22,23,24,25,26,27,28] or the *KCNE2* gene [29], which encodes the MiRP1 modulatory subunit of the *I*_f_ channel. Voltage-clamp experiments on wild-type and mutant human HCN4 channels expressed in COS-7, CHO and HEK-293 cells, as well as in *Xenopus* oocytes have revealed changes in the expression and/or kinetics of mutant HCN4 channels, but the extent to which especially the kinetic changes would affect *I*_f_ flowing during a human SAN action potential often remains unresolved.

In a previous review, we provided an overview of the *HCN4* and *KCNE2* mutations associated with sinus node dysfunction [30]. This overview was limited to the seven *HCN4* mutations and a single *KCNE2* mutation associated with sinus node dysfunction that were known at that time [2,19,20,21,22,23,24]. Today, however, there are as many as 15 newly discovered *HCN4* variants and mutations [25,26,27,28], some with clinical manifestations beyond sinus node dysfunction, and research is ongoing (e.g., [31]). Here, we first give an overview of these newly discovered *HCN4* variants and mutations and their effects on the characteristics of the HCN4 current. Next, we show how action potentials recorded from isolated human SAN pacemaker cells can be used as the command potential in simulated action potential clamp experiments to assess the effects of *HCN4* mutations on *I*_f_ flowing during a human SAN action potential. Furthermore, we point to apparent inconsistencies between data from clinical studies and data from *in vitro* and *in silico* experiments.

## 2. Mutations in *HCN4* and *KCNE2* Associated with Sinus Node Dysfunction

**Figure 1:**
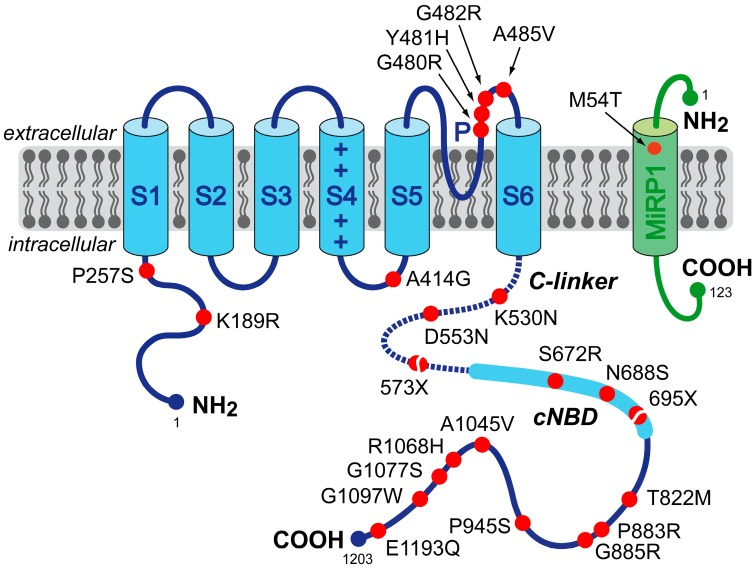
Schematic topology of the HCN4 and MiRP1 proteins. The HCN4 α-subunit has six transmembrane segments (S1–S6), a pore-forming loop (P) and intracellular *N*- and *C*-termini. The voltage sensor of the channel is formed by the positively-charged S4 helix. The *C*-terminus comprises the *C*-linker (dotted line) and the cyclic nucleotide-binding domain (cNBD), which is known to mediate cyclic AMP (cAMP)-dependent changes in HCN channel gating. The MiRP1 β-subunit has a single transmembrane segment with an extracellular *N*-terminus and intracellular *C*-terminus. Red dots indicate the location of the 23 known HCN4 and MiRP1 mutation sites associated with clinically established or potential sinus node dysfunction. The split dots indicate the truncations resulting from the 573X and 695X non-sense (truncating) mutations.

To date, 22 mutations or variants in *HCN4* and one in *KCNE2* have been associated with clinically established or potential sinus node dysfunction [2,19,20,21,22,23,24,25,26,27,28]. Figure 1 indicates the location of each of these mutations and variants on the HCN4 and MiRP1 proteins. Clinical observations, if reported, are summarized in Table 1, in the order of the mutations and variants on the protein. Changes in expression or kinetic properties associated with the mutations and variants are described below, in the same order, and summarized in Table 2. For completeness, previously reviewed mutations [30] are also included. Intriguingly, *HCN4* mutations are not only associated with sinus node dysfunction, but also with AF, left ventricular non-compaction cardiomyopathy (LVNC) and atrioventricular (AV) block.

In our overview, we did not include the *HCN4* mutation in a Brugada syndrome patient described by Ueda *et al.* [32]. The insertion of four bases at a splicing junction of exon 2 and intron 2 likely results in a defective HCN4 protein without the ability to form functional tetramers. A functional analysis in COS-7 cells demonstrated that almost equal amounts of normal and abnormal splicing products were expressed, but *in vitro* electrophysiological data are not available [32]. Furthermore, we did not include the “likely pathogenic” A195V and V759I variants in *HCN4* that were identified by Evans *et al.* [33] in two cases of sudden infant death syndrome (SIDS), because both clinical and *in vitro* data on the effects of these variants are lacking.

A recent abstract mentioned five novel *HCN4* mutations in patients suffering from sinus node dysfunction, dyspnea and AF [31]. Unfortunately, neither the exact mutations nor their effects on the HCN4 current were specified. Thus, these mutations could not be included in our overview.

**Table 1:** Clinical observations in carriers of mutations in *HCN4* or *KCNE2*.

Mutation	Mutation Carriers	Clinical Presentation	Study	
**Mutations in *HCN4***			
P257S	single index patient (65-year-old male)	diagnosed with paroxysmal AF at age 29; AF became permanent at age 43 years; sinus node dysfunction during ajmaline test in proband and proband’s father; 73 pauses >2.0 s on 24-h Holter monitoring	Macri *et al.* [26]
A414G	3 members of a single family	AF and LVNC in 74-year-old male index patient; combined sinus bradycardia and LVNC in his two mutation-carrying sons; severe sinus bradycardia involving 12 episodes of standstill on Holter monitoring in one of the sons	Milano *et al.* [27]
G480R	8 members of a single family	asymptomatic sinus bradycardia from a young age, with normal chronotropic and exercise capacity; minimum, average and maximum heart rates of 31 ± 8, 48 ± 12 and 101 ± 21 beats/min, respectively, in the 8 mutation carriers *vs.* 55 ± 9, 73 ± 11 and 126 ± 16 beats/min, respectively, in the 8 non-carriers	Nof *et al.* [21]
Y481H	4 members of two families with a common ancestral haplotype	combined sinus bradycardia and LVNC; frequent episodes of severe bradycardia (heart rate < 30 beats/min) and pacemaker implantation in the index patient of the first family; severe sinus bradycardia (40 beats/min) in the index patient of the second family and pacemaker implantation in his mutation-carrying mother because of bradyarrhythmias	Milano *et al.* [27]
G482R	6 members of a single family	combined sinus bradycardia and LVNC; pacemaker implanted in three mutation carriers because of bradycardia-related symptoms (average heart rate of 46 beats/min); MVP in two individuals	Milano *et al.* [27]
G482R	3 members of a single German family	combined sinus bradycardia, LVNC, and MVP; minimum and average heart rates of 21 and 34 beats/min, respectively, and pacemaker implantation in the index patient	Schweizer *et al.* [28]
A485V	14 members of three Moroccan Jewish decent families	symptomatic familial sinus bradycardia with normal chronotropic and exercise capacity; minimum, average and maximum heart rates of 37 ± 3, 58 ± 6 and 117 ± 27 beats/min in the 14 mutation carriers, respectively, *vs.* 49 ± 11, 77 ± 12 and 140 ± 32 beats/min, respectively, in the 6 non-carriers	Laish-Farkash *et al.* [23]
K530N	6 members of a single family	mild, asymptomatic sinus bradycardia (50–60 beats/min) in the index patient; familial age-dependent tachycardia-bradycardia syndrome and persistent AF; no AF or any other relevant cardiac arrhythmia in non-carriers	Duhme *et al.* [24]
D553N	single index patient (43-year-old female) and two family members	wide spectrum of cardiac arrhythmias, including severe bradycardia (24-h average of 39 beats/min), QT prolongation and *Torsade de Pointes* in the index patient; QT prolongation in family members	Ueda *et al.* [19]
573X	single index patient (66-year-old female)	idiopathic sinus bradycardia of 41 beats/min; chronotropic incompetence; intermittent episodes of AF	Schulze-Bahr *et al.* [2]
S672R	15 members of a single Italian family	asymptomatic sinus bradycardia; average resting heart rate, corrected for age and gender, of 52.2 ± 1.4 beats/min (range 43–60 beats/min), in the 15 mutation carriers *vs.* 73.2 ± 1.6 beats/min (range 64–81 beats/min) in the 12 non-affected family members	Milanesi *et al.* [20]
695X	8 members of a single German family	marked sinus bradycardia with no signs of chronotropic incompetence; basal heart rate of 45.9 ± 4.6 beats/min (range 38–51 beats/min) in the 8 mutation carriers *vs.* 66.5 ± 9.1 beats/min in the 6 non-carriers; minimum heart rate of 35.9 ± 5.6 *vs.* 47.2 ± 5.9 beats/min; maximum heart rates of 160.3 ± 12.6 *vs.* 171.8 ± 18.7 beats/min; LVNC in 5 of the mutation carriers	Schweizer *et al.* [22,28]
P883R	single male patient	sinus bradycardia (35 to 40 beats/min), paroxysmal AF (tachycardia-bradycardia syndrome) and LVNC; pacemaker implantation	Schweizer *et al.* [28]
G1097W	single index patient (69-year-old male)	complete AV block with wide QRS, but no sinus nodal dysfunction; atrial rate of 132 beats/min; ventricular rate of 33 beats/min; pacemaker implantation at the age of 51 years	Zhou *et al.* [25]
**Mutations in *KCNE2***			
M54T	single index patient (55-year-old Caucasian male)	history of marked sinus bradycardia; average heart rate of 43 beats/min (range 30–125 beats/min), along with pauses; daughter died suddenly at the age of 13, and post-mortem genetic testing revealed the M54T mutation	Nawathe *et al.* [29]

Mutations are heterozygous with autosomal dominant inheritance. AF, atrial fibrillation; LVNC, left ventricular non-compaction cardiomyopathy; MVP, mitral valve prolapse; QT prolongation, prolongation of the rate-corrected QT interval on the electrocardiogram.

### 2.1. HCN4-K189R

Macri *et al.* [26] sought to determine if genetic variation in the coding region of *HCN4* is associated with AF. They identified seven novel HCN4 variants in their cohort of 527 unrelated individuals with early-onset AF, as well as three novel variants in their cohort of 443 referent individuals without history or ECG evidence of AF. K189R, located in the *N*-terminus (Figure 1), is one of the HCN4 variants identified in the AF cases. Macri *et al.* [26] overexpressed wild-type and homomeric K189R variant HCN4 channels in CHO cells and carried out voltage-clamp experiments. They did not observe differences in characteristics of the wild-type and variant HCN4 current (Table 2). Of note, Macri *et al.* [26] did not study heteromeric wild-type/K189R channels, which would have better resembled the heterozygous condition in individuals carrying the K189R variant.

**Table 2:** Effect of mutations and variants in *HCN4* or *KCNE2* on HCN4 current.

Mutation	Type of Expression	Expression System	Shift in V_1/2_ or Activation Threshold (mV)	Slope Factor (mV)	Time Constant of Activation	Time Constant of Deactivation	Reversal Potential	Full-Activated Current Density	Channel Expression	Sensitivity to cAMP	Reference
**Mutations and Variants in *HCN4***											
K189R	homomeric	CHO	=	=	?	?	?	=	?	?	Macri *et al.* [26]
P257S	heteromeric	CHO	=	=	?	?	?	=	≈50%	?	Macri *et al.* [26]
	homomeric	CHO						0%	↓		Macri *et al.* [26]
A414G	heteromeric	CHO	−23.9	=	?	?	?	=	?	?	Milano *et al.* [27]
G480R	heteromeric	oocyte, HEK	≈−15	?	↑ ^a^	?	=	≈50%	?	= ^b^	Nof *et al.* [21]
	homomeric	oocyte, HEK	≈−30	?	↑ ^a^	?	=	≈12%	↓	= ^b^	Nof *et al.* [21]
		oocyte	?	?	?	?	?	≈20%	?	?	Laish-Farkash *et al.* [23]
Y481H	heteromeric	CHO	−43.9	=	?	?	?	=	?	?	Milano *et al.* [27]
G482R	heteromeric	HEK	=	=	=	=	=	35%	=	?	Schweizer *et al.* [28]
		CHO	−38.7	=	?	?	?	=	?	?	Milano *et al.* [27]
	homomeric	HEK	?	?	?	?	?	6%	=	?	Schweizer *et al.* [28]
A485V	heteromeric	oocyte, HEK	≈−30	?	↑ ^a^	↑ ^a^	=	≈33%	=	?	Laish-Farkash *et al.* [23]
	homomeric	oocyte, HEK	≈−60	?	↑ ^a^	↑ ^a^	=	≈5%	=	↓	Laish-Farkash *et al.* [23]
K530N	heteromeric	HEK	≈−14	=	237%	=	=	=	?	↑	Duhme *et al.* [24]
	homomeric	HEK	=	=	=	=	=	=	?	=	Duhme *et al.* [24]
D553N	heteromeric	COS	=	=	≈90%	≈110%	?	≈37%	↓	?	Ueda *et al.* [19]
		oocyte, COS	?	?	?	?	?	≈54%	=	?	Netter *et al.* [34]
	homomeric	COS	=	=	≈90%	≈110%	?	≈8%	↓	?	Ueda *et al.* [19]
		oocyte, COS	=	=	=	=	?	≈12%	=	↓	Netter *et al.* [34]
573X	heteromeric	COS	= ^c^	−1.9 ^c^	=	?	?	?	=	↓	Schulze-Bahr *et al.* [2]
	homomeric	COS	−4.6 ^c^	=	=	? ^a^	?	?	=	↓	Schulze-Bahr *et al.* [2]
S672R	heteromeric	HEK	−4.9	=	=	≈74%	?	?	?	?	Milanesi *et al.* [20]
	homomeric	HEK	−8.4	=	=	≈63%	?	?	?	=	Milanesi *et al.* [20]
		oocyte	−6.1	?	≈180%	≈90%	?	?	?	↓	Xu *et al.* [32]
N688S	homomeric	CHO	=	=	?	?	?	=	?	?	Macri *et al.* [26]
695X	heteromeric	HEK	=	=	=	?	?	=	?	↓	Schweizer *et al.* [22]
	homomeric	HEK	=	−3.5	72%	=	=	?	?	↓	Schweizer *et al.* [22]
T822M	homomeric	CHO	=	=	?	?	?	=	?	?	Macri *et al.* [26]
G885R	homomeric	CHO	=	=	?	?	?	=	?	?	Macri *et al.* [26]
P945S	homomeric	CHO	=	=	?	?	?	=	?	?	Macri *et al.* [26]
A1045V	homomeric	CHO	=	=	?	?	?	=	?	?	Macri *et al.* [26]
R1068H	homomeric	CHO	=	=	?	?	?	=	?	?	Macri *et al.* [26]
G1077S	homomeric	CHO	=	=	?	?	?	=	?	?	Macri *et al.* [26]
G1097W	heteromeric	CHO	−7.6	=	?	81%	?	55%	?	?	Zhou *et al.* [25]
	homomeric	CHO	−12	=	?	79%	?	47%	?	=	Zhou *et al.* [25]
E1193Q	homomeric	CHO	=	=	?	?	?	=	?	?	Macri *et al.* [26]
**Mutations in* KCNE2***											
M54T	homomeric	NRVM	=	=	192%	=	?	18%	?	?	Nawathe *et al.* [29]

Data are the changes relative to wild-type current. ?, not reported; ≈, estimated from presented figures; ↓, decreased; ↑, increased; =, unchanged. ^a^ Changes reported, but no quantitative data provided; ^b^ performed in oocytes, which lack cAMP modulation, due to high basal activity [21]; ^c^ with 15-s hyperpolarizing pulses (at 20–22 °C); oocyte, HEK, COS and NRVM: *Xenopus* oocytes, HEK-293 cells, COS-7 cells and neonatal rat ventricular cardiomyocytes, respectively.

### 2.2. HCN4-P257S

Like the above K189R variant, P257S, also located in the *N*-terminus (Figure 1), is one of the seven HCN4 variants identified by Macri *et al.* [26] in their cohort of individuals with early-onset AF. Expression of homomeric P257S-variant HCN4 channels in CHO cells did not result in any measurable HNC4 current in voltage-clamp experiments, whereas co-expression of wild-type and variant HCN4 did not reveal any changes with respect to wild-type HNC4 current (Table 2). However, with the use of immunocytochemistry and confocal microscopy, Macri *et al.* [26] also demonstrated that P257S variant channels were not detectable at the plasma membrane, but instead were retained in the cytoplasm, which suggests that the P257S variant disrupts trafficking to the cell membrane. Macri *et al.* [26] further demonstrated that, when co-expressed, wild-type HCN4 channels do not colocalize with P257S variant channels at the cell membrane. The latter findings suggest, despite the similar current density in voltage-clamp experiments (Table 2), that the P257S variant may be related to the clinically observed AF (Table 1) as a result of haploinsufficiency, with only the single wild-type allele contributing to the production of the HCN4 channel protein and current.

### 2.3. HCN4-A414G

A414G is a mutation in the S4–S5 linker (Figure 1) associated with sinus bradycardia, AF and LVNC in three members of a single family (Table 1). Functional analysis in CHO cells revealed a significant hyperpolarizing shift in the voltage dependence of activation of heteromerically expressed A414G mutant HCN4 channels (Table 2). This shift resulted in a dramatic decrease of the fully-activated HCN4 current density in the voltage range of the diastolic depolarization of SAN cells [27].

### 2.4. HCN4-G480R

G480R is one of the currently known mutations in the pore-forming loop of the HCN4 protein (Figure 1). It is associated with asymptomatic sinus bradycardia from a young age, with normal chronotropic and exercise capacity (Table 1). Western blot analysis demonstrated significantly reduced membrane expression of homomeric HCN4-G480R channels in HEK-293 cells [21]. Functional analysis in *Xenopus* oocytes and HEK-293 cells revealed a decrease in fully-activated current density, accompanied by a hyperpolarizing shift in the voltage dependence of activation and slowing of activation kinetics (Table 2). In *Xenopus* oocytes, neither wild-type nor G480R currents were modulated by β-adrenergic regulation, likely due to the high levels of endogenous cyclic AMP (cAMP) in *Xenopus* oocytes [35]. Thus, whether the G480R mutation affects the sensitivity to cAMP is unresolved. Laish-Farkash *et al.* [23] later confirmed the decrease of fully-activated current density in HEK-293 cells.

### 2.5. HCN4-Y481H

Another, recently discovered mutation in the pore-forming loop of the HCN4 protein is Y481H (Figure 1). It is associated with combined sinus bradycardia and LVNC in four members of two families with a common ancestral haplotype (Table 1). If heteromerically expressed in CHO cells, the Y481H mutation results in a >40-mV shift in the voltage dependence of activation towards more negative potentials (Table 2). As a consequence, the fully-activated HCN4 current density in the voltage range of diastolic depolarization of SAN cells is almost zero [27].

### 2.6. HCN4-G482R

Recently, both Milano *et al.* [27] and Schweizer *et al.* [28] reported an association of the G482R mutation with combined sinus bradycardia and LVNC in a total of nine individuals from two families (Table 1). Like the above G480R and Y481H mutations, the G482R mutation is located in the pore-forming loop of the HCN4 protein (Figure 1). Patch-clamp experiments in HEK-293 cells by Schweizer *et al.* [28] demonstrated that homomeric G482R mutant channels were non-functional, despite similar surface expression of HCN4 wild-type and mutant subunits. In case of heteromeric expression, the mutation exerted dominant negative effects as revealed by a 65% reduction in fully-activated current density, without affecting the voltage dependence of activation. Milano *et al.* [27], on the other hand, reported a strong hyperpolarizing shift in the voltage dependence of activation of G482R channels that were heteromerically expressed in CHO cells, resulting in a negligible fully-activated HCN4 current density in the voltage range of diastolic depolarization of SAN cells.

### 2.7. HCN4-A485V

Laish-Farkash *et al.* [23] observed familial sinus bradycardia in 14 members of three Moroccan Jewish decent families carrying the A485V mutation, which is another mutation in the pore-forming loop (Figure 1). Western blot analysis revealed significantly reduced membrane expression of homomeric A485V mutant channels in HEK-293 cells [23]. Functional analysis of both homomerically and heteromerically expressed mutant channels in *Xenopus* oocytes and HEK-293 cells demonstrated large hyperpolarizing shifts of the voltage dependence of activation, slowing of both activation and deactivation, and a reduction in fully-activated current density (Table 2).

### 2.8. HCN4-K530N

Tachycardia-bradycardia syndrome and persistent AF were observed in six members of a single family carrying the K530N mutation in *HCN4* [24], located in the *C*-linker of the HCN4 protein (Figure 1). Patch-clamp experiments in HEK-293 cells revealed similar characteristics of wild-type channels and homomerically expressed mutant channels (Table 2). However, experiments on heteromerically expressed channels demonstrated a hyperpolarizing shift of the half-maximal activation voltage and slowed activation of the HCN4 current. Furthermore, heteromeric channels showed a larger sensitivity to cAMP than either homomeric mutant or wild-type channels, as demonstrated by the ≈7-mV larger cAMP-induced shift of the activation curve and the larger change in the activation time constant.

### 2.9. HCN4-D553N

Also located in the *C*-linker is the D553N mutation (Figure 1). Several electrophysiological abnormalities, including severe bradycardia and QT prolongation (*i.e.*, prolongation of the rate-corrected QT interval on the electrocardiogram), were noticed in three members of a single family carrying this mutation [24]. A functional study in COS-7 cells [19] showed a reduced membrane expression and decreased current, because of a dominant-negative trafficking defect of the D553N mutant protein. The voltage dependence of activation of the mutant HCN4 channel was comparable to the wild-type, but activation was slightly faster, while deactivation was slightly slower (Table 2). On the other hand, Netter *et al.* [34] reported that D553N mutant channels have normal trafficking, with similar surface expression of D553N and wild-type channels in COS-7, HeLa and HL-1 cells. In both *Xenopus* oocytes and COS-7 cells, D553N channels generated currents with reduced amplitude, but unaltered kinetics. Furthermore, homomeric D553N channels did not respond to adrenergic stimulation.

### 2.10. HCN4-573X

In a single index patient, Schulze-Bahr *et al.* [2] observed sinus bradycardia with chronotropic incompetence and episodes of AF (Table 1). This patient carried the 573X non-sense (truncating) mutation in the *C*-linker (Figure 1). The mutation thus resulted in a truncated HCN4 protein that lacks the cyclic nucleotide-binding domain (cNBD), which mediates the cAMP effects on gating of the HCN4 channel. In COS-7 cells, mutant subunits showed normal intracellular trafficking and integration into the cell membrane [2]. Patch-clamp experiments demonstrated a steeper steady-state activation curve with a shift to more hyperpolarized potentials, but this required excessively long hyperpolarizing voltage steps to become apparent (Table 2). Both homomeric and heteromeric channels appeared insensitive to cAMP, demonstrating a dominant-negative effect of the mutant on wild-type subunits.

### 2.11. HCN4-S672R

Fifteen carriers of the S672R mutation, all members of a single Italian family, showed asymptomatic sinus bradycardia (Table 1). A functional study in HEK-293 cells revealed a shift in channel activation to more hyperpolarized potentials and faster deactivation of both homomeric and heteromeric mutant channels (Table 2). The cAMP-dependent shifts in voltage dependence of activation, as assessed in inside-out macropatches, were similar in wild-type and homomeric mutant channels [20], suggesting that the S672R mutation did not affect the sensitivity to cAMP, notwithstanding its location in the cNBD (Figure 1). Xu *et al.* [36], on the other hand, made inside-out patch-clamp recordings in *Xenopus* oocytes and found a reduced sensitivity to cAMP, which, however, was challenged in a review by DiFrancesco [7].

### 2.12. HCN4-N688S

The N688S variant is also located in the cNBD of the HCN4 subunit (Figure 1). It is one of the three novel variants that Macri *et al.* [26] observed in their cohort of 443 referent individuals without history or ECG evidence of AF. In patch-clamp experiments on CHO cells, no functional effects of the N688S variant were observed (Table 2). Of note, preservation of cAMP dependence was not assessed.

### 2.13. HCN4-695X

In a single German family, Schweizer *et al.* [22] observed marked sinus bradycardia without signs of chronotropic incompetence in eight carriers of the 695X non-sense mutation in *HCN4* (Table 1). Recently, Schweizer *et al.* [28] reported LVNC in five of the eight mutation carriers. Like the above 573X mutation, the 695X mutation results in a truncated cNBD (Figure 1). Patch-clamp experiments in HEK-293 cells demonstrated a steeper slope of the activation curve and faster activation of homomeric 695X mutant current, as well as insensitivity to cAMP (Table 2). Heteromeric channels failed to respond to cAMP, like homomeric mutant channels, indicating a dominant-negative suppression of cAMP responsiveness by the mutant subunits, notwithstanding the apparent absence of signs of chronotropic incompetence in the mutation carriers.

### 2.14. HCN4-T822M

The T822M variant, located in the distal *C*-terminus beyond the cNBD (Figure 1), is one of the seven novel variants that Macri *et al.* [26] observed in their cohort of 527 unrelated individuals with early-onset AF. In patch-clamp experiments on CHO cells, no functional effects of the T822M variant were observed (Table 2).

### 2.15. HCN4-P883R

The P883R mutation in *HCN4* was found by Schweizer *et al.* [28] in a single patient who showed sinus bradycardia, tachycardia-bradycardia syndrome and LVNC and who required pacemaker implantation. Unfortunately, patch-clamp data on the P883R mutant channel are not available. The mutation is therefore not included in Table 2.

### 2.16. HCN4-G885R, -P945S, -A1045V, -R1068H and -G1077S

Like the T822M variant, the G885R, P945S, A1045V, R1068H and G1077S variants, which are all located in the distal *C*-terminus beyond the cNBD (Figure 1), are novel HCN4 variants that Macri *et al.* [26] observed in their cohort of 527 unrelated individuals with early-onset AF (G885R, P945S and G1077S) or in their cohort of 443 referent individuals without history or ECG evidence of AF (A1045V and R1068H). In patch-clamp experiments on CHO cells, none of the five variants showed functional effects (Table 2).

### 2.17. HCN4-G1097W

Zhou *et al.* [25] observed the G1097W mutation in a single index patient with AV block, but not sinus nodal dysfunction (Table 1). In patch-clamp experiments on CHO cells, both homomeric and heteromeric channels demonstrated a hyperpolarizing shift in the voltage dependence of activation, a reduced fully-activated current density and a faster deactivation (Table 2). The sensitivity to intracellular cAMP, as assessed for homomeric channels, was not affected.

### 2.18. HCN4-E1193Q

The E1193Q variant, located near the end of the *C*-terminus of the HCN4 protein (Figure 1), is one of the seven novel HCN4 variants that Macri *et al.* [26] observed in their cohort of 527 unrelated individuals with early-onset AF. Patch-clamp experiments on CHO cells did not reveal any functional effects of the variant (Table 2).

### 2.19. KCNE2-M54T

Nawathe *et al.* [29] reported marked sinus bradycardia, along with pauses, in a single index patient who carried the M54T mutation in *KCNE2* (Table 1). The mutation is in the transmembrane segment of the *KCNE2*-encoded MiRP1 protein (Figure 1). Patch-clamp experiments in neonatal rat ventricular myocytes demonstrated that co-expression with M54T MiRP1 decreased HCN4 current density by >80% compared to HCN4 alone or HCN4 co-expressed with wild-type MiRP1. Furthermore, co-expression with M54T MiRP1 slowed HCN4 activation at physiologically relevant voltages, while HCN4 deactivation and the voltage dependence of activation were not affected (Table 2).

## 3. Functional Effects of Novel *HCN4* Mutations on Human *I*_f_

In our previous review [30], we assessed the functional effects of the then known *HCN4* and *KCNE2* mutations on human *I*_f_ through simulated action potential clamp experiments. Action potentials recorded from single, isolated human SAN cells [37] were used as command potentials, and *I*_f_ was simulated using mathematical equations based on the voltage clamp data that we had acquired from single, isolated human SAN cells [38,39]. Thus, we reconstructed *I*_f_ during the time course of a human SAN action potential. Here, we apply this approach to the aforementioned P257S, A414G, Y481H, G482R and G1097W mutations in *HCN4*. We selected these five mutations, because they were not included in our previous review, they have clinically established effects (Table 1), and patch-clamp data on heteromerically expressed mutant channels, resembling the heterozygous mutation carrier situation, are available (Table 2).

### 3.1. Numerical Reconstruction of I_f_

**Figure 2:**
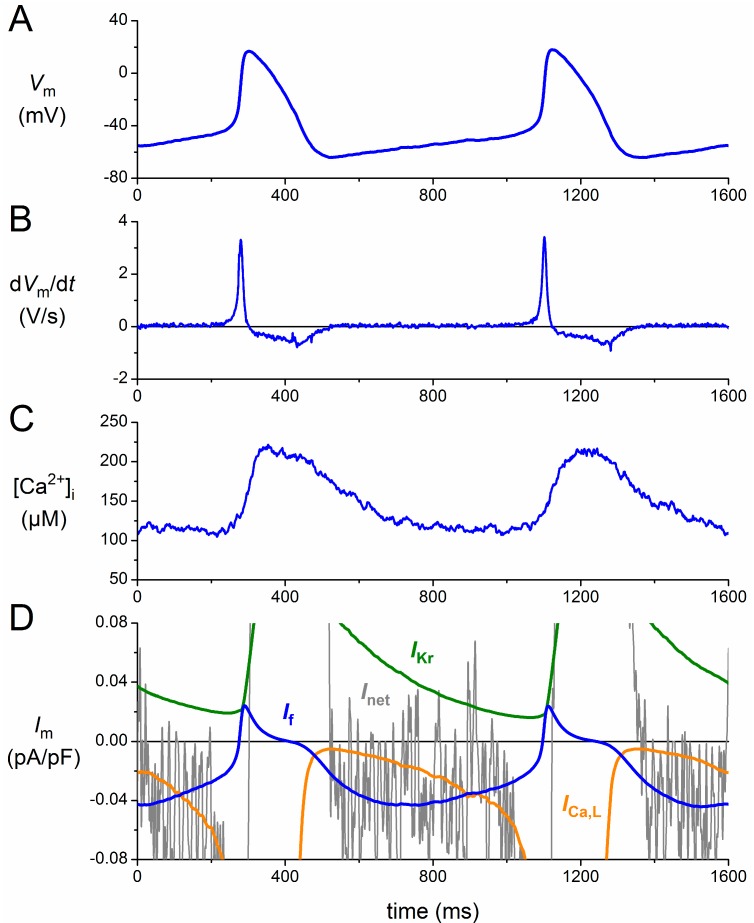
Membrane currents of a human sinoatrial node (SAN) pacemaker cell assessed by a simulated action potential clamp. (**A**) Action potentials recorded from a human SAN pacemaker cell used for the action potential clamp; (**B**) associated rate of change of the membrane potential (d*V*_m_/d*t*); (**C**) global intracellular calcium concentration ([Ca^2+^]_i_) in a different cell with a highly similar cycle length; and (**D**) numerically reconstructed membrane current (*I*_m_): L-type calcium current (*I*_Ca,L_), *I*_f_, delayed rectifier potassium current (*I*_Kr_) and net membrane current (*I*_net_). See the text for details.

Figure 2A shows the action potentials that were used, as part of a train of action potentials, in the simulated action potential clamp experiments. The associated rate of change of the membrane potential (d*V*_m_/d*t*) is shown in Figure 2B, whereas a recording of the global intracellular calcium concentration ([Ca^2+^]_i_), from a different cell with virtually the same cycle length [40], is shown in Figure 2C. With these data, the ion current traces of Figure 2D could be reconstructed, focusing on the diastolic depolarization phase. The noisy grey trace shows the net membrane current (*I*_net_), as computed from *I*_net_ = −*C*_m_ × d*V*_m_/d*t*, where d*V*_m_/d*t* is taken from Figure 2B, and *C*_m_ and *V*_m_ denote the membrane capacitance and membrane potential, respectively. The orange and green traces of Figure 2D show the L-type calcium current (*I*_Ca,L_) and delayed rectifier potassium current (*I*_Kr_), respectively, which, together with *I*_f_ (blue trace), supposedly constitute the main voltage-dependent ion currents during diastolic depolarization. Figure 2D illustrates the importance of *I*_f_ as a pacemaker current, generating an inward current during diastolic depolarization of similar amplitude as *I*_net_. It should, however, be kept in mind that *I*_net_ is the net result of multiple inward and outward currents, including *I*_Ca,L_ and *I*_Kr_, which may interact in a complex manner [4].

For the numerical reconstruction of *I*_Ca,L_ and *I*_Kr_, we followed the approach of Chandler *et al.* [11], who used the equations of the Courtemanche *et al.* [41] human atrial cell model, scaled down by 32% and 55%, respectively, in accordance with the relative abundance (human sinus node *vs.* human right atrium) of mRNAs responsible for the *I*_Ca,L_ and *I*_Kr_ ion channels. *I*_Kr_ could be reconstructed with the train of action potentials of Figure 2A, but for the reconstruction of *I*_Ca,L_, we also needed the [Ca^2+^]_i_ data of Figure 2C, because the inactivation kinetics of this current are not only voltage dependent, but also calcium dependent. For the numerical reconstruction of *I*_f_, we used the mathematical equations that we previously derived from the voltage clamp data that we had acquired from single, isolated human SAN pacemaker cells [38,39].

**Figure 3:**
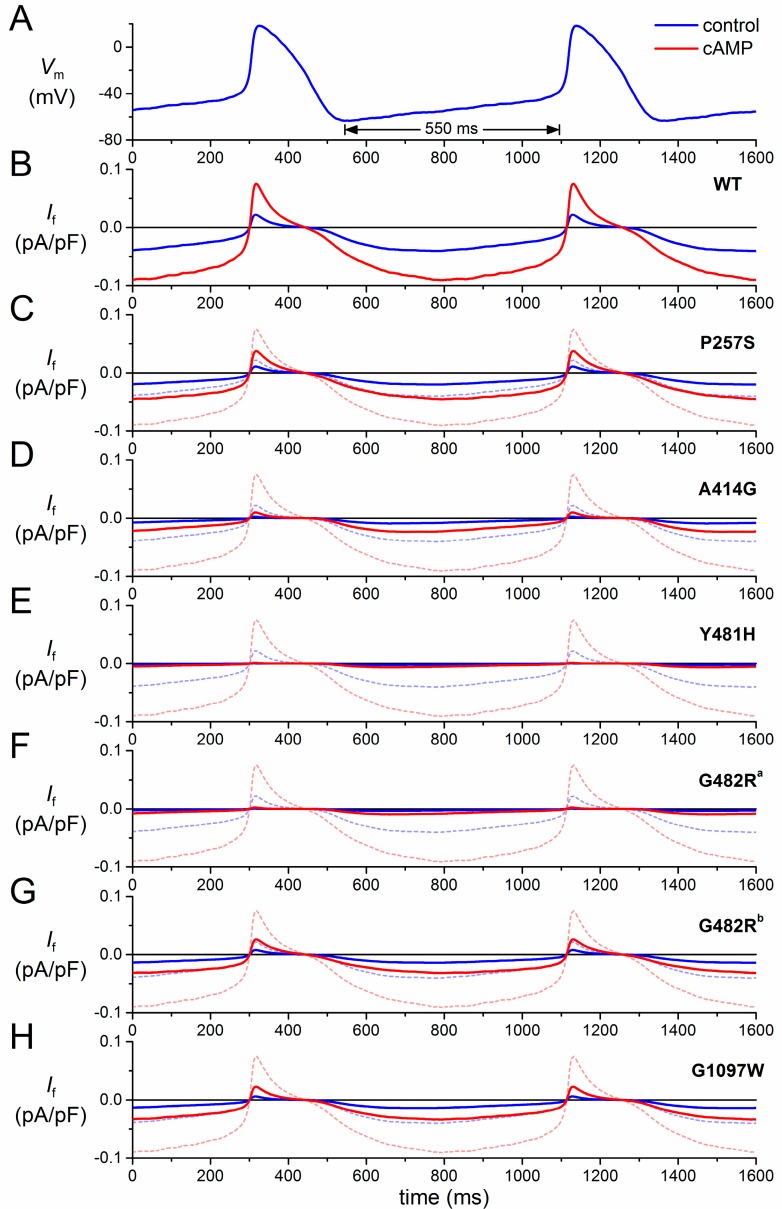
Effect of mutations in *HCN4* on *I*_f_ in a human SAN pacemaker cell assessed by simulated action potential clamp. (**A**) Action potentials recorded from a human SAN pacemaker cell used for action potential clamp; (**B**) computed wild-type (WT) *I*_f_ of a human SAN pacemaker cell during the action potentials of (**A**) under control conditions (“control”, blue line) and upon adrenergic stimulation (“cAMP”, red line); (**C**–**H**) computed *I*_f_ of a human SAN pacemaker cell carrying heterozygous mutation in *HCN4*, as indicated, during the action potentials of (**A**) under control conditions (solid blue line) and upon adrenergic stimulation (solid red line). Wild-type *I*_f_ of (**B**) under control conditions (dashed blue line) and upon adrenergic stimulation (dashed red line) are shown for reference. G482R traces, labelled “a” and “b”, are based on data from Milano *et al.* [27] and Schweizer *et al.* [28], respectively.

Figure 3 shows the effects of mutations in *HCN4* on *I*_f_, both under control conditions (blue traces) and upon adrenergic stimulation (red traces). In the absence of action potentials recorded upon adrenergic stimulation, the same train of action potentials (Figure 3A) was used to reconstruct *I*_f_ in the case of elevated cAMP levels. This was achieved by repeating the simulations with the activation and time constant curves shifted by +15 mV [30]. The resulting *I*_f_ trace is shown as a solid red line in Figure 3B. It is immediately apparent from Figure 3B that the amplitude of *I*_f_ is more than doubled upon adrenergic stimulation.

**Figure 4:**
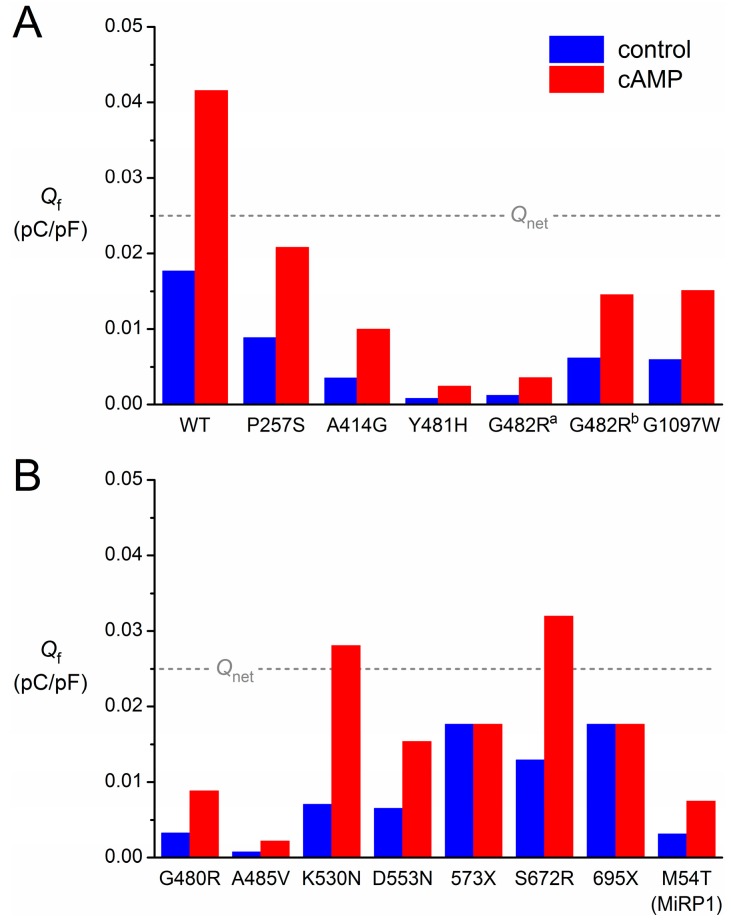
Contribution of *I*_f_ to diastolic depolarization for each of the heterozygous mutations in *HCN4* or *KCNE2*. The charge carried by *I*_f_ (*Q*_f_) during the 25-mV, 550-ms spontaneous depolarization from the maximum diastolic potential of −63 mV of the human SAN action potential is as indicated in Figure 3A. The blue bars are computed from the *I*_f_ traces under control conditions (“control”). The red bars are computed from the *I*_f_ traces upon adrenergic stimulation (“cAMP”). The dashed grey line indicates the charge of 0.025 pC/pF carried by the net membrane current (*Q*_net_) during the 25-mV depolarization. (**A**) Mutations of Figure 3; (**B**) mutations assessed in previous publication [30].

To quantify the contribution of *I*_f_ to diastolic depolarization, we computed the charge carried by *I*_f_ (*Q*_f_) during the 25-mV, 550-ms depolarization that starts at the maximum diastolic potential of −63 mV (Figure 3A, double-headed arrow). Under control conditions, *Q*_f_ amounts to 0.018 pC/pF (Figure 4, leftmost blue bar), which is somewhat smaller than the net charge flow of 0.025 pC/pF or, equivalently, 25 mV (Figure 4, dashed grey line). Upon adrenergic stimulation, *Q*_f_ increases 2.4-fold to 0.042 pC/pF (Figure 4, leftmost red bar). Thus, one might say that wild-type *I*_f_ has a “depolarization reserve” of 24 mV, *i.e.*, the difference between the “depolarization power” of 18 mV under basal conditions and 42 mV upon adrenergic stimulation.

To simulate the effects of the mutations, we adapted the *I*_f_ parameter settings for the mutation of interest according to the data presented in Table 2. Thus, the P257S mutation is simulated through a 50% reduction in the fully-activated conductance of *I*_f_, whereas the A414G mutation is simulated through a −23.9 mV shift in its voltage dependence, *etc*. The parameter settings for each of the aforementioned novel mutations in *HCN4*, all expressed as changes relative to the wild-type, are listed in Table 3, together with the parameter settings that we applied in our previous review to simulate the then known mutations in *HCN4* and *KCNE2*.

### 3.2. HCN4-P257S

In the case of the P257S mutation, there is no shift in voltage dependence. Furthermore, sensitivity to cAMP is not affected. However, the current density of heteromeric P257S channels is reduced to ≈50% (Table 2). Accordingly, we reduced the fully-activated conductance of *I*_f_ to 50% of the wild-type, which of course leads to an equivalent reduction in the *I*_f_ amplitude (Figure 3C) and *Q*_f_ (Figure 4A), both under control conditions and upon adrenergic stimulation. One may question whether these relatively mild functional effects are the main cause of the AF and (hidden) sinus node dysfunction observed in the single index patient.

### 3.3. HCN4-A414G

In the case of the A441G mutation, there is a >20-mV hyperpolarizing shift in the voltage dependence of the *I*_f_ kinetics. As a result, *I*_f_ is significantly reduced throughout diastole (Figure 3D), and the charge carried by *I*_f_ is reduced to 20% of the wild-type under control conditions and 24% upon adrenergic stimulation (Figure 4A). These functional effects may underlie the sinus bradycardia observed in the mutation-carrying family (Table 1).

**Table 3:** Parameter settings in simulated action potential clamp experiments.

Mutation	Scaling Factor for *I*_f_ Conductance	Shift (mV)	Shift with cAMP (mV)
*HCN4*-P257S	0.50	0	+15
*HCN4*-A414G	1	−23.9	−8.9
*HCN4*-G480R	0.50	−15	0
*HCN4*-Y481H	1	−43.9	−28.9
*HCN4*-G482R ^a^	1	−38.7	−23.7
*HCN4*-G482R ^b^	0.35	0	+15
*HCN4*-A485V	0.33	−30	−15
*HCN4*-K530N	1	−14	+7.8
*HCN4*-D553N	0.37	0	+15
*HCN4*-573X	1	0	0
*HCN4*-S672R	1	−4.9	+10.1
*HCN4*-695X	1	0	0
*HCN4*-G1097W	0.55	−7.6	+7.4
*KCNE2*-M54T	0.18	0	+15

Scaling factor and shifts relative to the wild-type. Shifts applied to both the steady-state activation curve and time constant curve. ^a^ According to Milano *et al.* [27]; ^b^ according to Schweizer *et al.* [28].

### 3.4. HCN4-Y481H

*I*_f_ is dramatically reduced in the case of the Y481H mutation, which causes a >40-mV hyperpolarizing shift in the voltage dependence of the *I*_f_ kinetics (Figure 3E). This is reflected in the charge carried by *I*_f_ during diastole, which amounts to only 5%–6% of the wild-type (Figure 4A). This dramatic functional effect seems compatible with the severe bradycardia in the Y481H mutation carriers (Table 1).

### 3.5. HCN4-G482R

A severe bradycardia is also observed in carriers of the G482R mutation (Table 1). In line, *I*_f_ is dramatically reduced (Figure 3F), with a *Q*_f_ of only 7%–9% of the wild-type (Figure 4A), if we base our simulations on the patch-clamp data by Milano *et al.* [27], which show a very strong hyperpolarizing shift in the voltage dependence of *I*_f_. However, patch-clamp data on the same mutation by Schweizer *et al.* [28] do show a 65% reduction in the fully-activated conductance of *I*_f_, rather than a shift in its voltage dependence. If we base our simulations on the latter data, we of course obtain a 65% reduction in *I*_f_ amplitude (Figure 3G) and *Q*_f_ (Figure 4A), both under control conditions and upon adrenergic stimulation.

### 3.6. HCN4-G1097W

With the combined effect of a shift in voltage dependence and a 45% reduction in fully-activated conductance, the net effect of the G1097W mutation (Figure 3H) is highly similar to the net effect of a 65% reduction in fully-activated conductance *per se* (G482R mutation, Figure 3G). Actually, the charge carried by *I*_f_ is reduced to ≈35% in either case (Figure 4A). Yet, no sinus node dysfunction is reported for the G1097W mutation [25]. However, such a dysfunction may have become obscured by the apparent 4:1 AV block in the single index patient (Table 1).

### 3.7. Limitations in the Reconstruction of I_f_

The above reconstruction of *I*_f_ comes with several limitations. First, we used a single predetermined action potential waveform, with an identical cycle length and identical duration of diastolic depolarization for both wild-type and mutant *I*_f_ reconstructions. Furthermore, this single waveform was used to reconstruct *I*_f_ under control conditions, as well as upon adrenergic stimulation. We did so because recordings of human SAN action potentials upon adrenergic stimulation or with mutant *I*_f_ channels are nonexistent. In a preliminary study [42], we varied the cycle length of human SAN action potential waveforms between 500 and 1500 m by manipulating the rate of diastolic depolarization and used these manipulated action potential waveforms in action potential clamp experiments on undifferentiated human cardiac myocyte progenitor cells. We found that the amplitude of HCN4 current expressed in these cells increased with increasing cycle length. Thus, the longer cycle lengths in bradycardic patients may partially counteract the *I*_f_ reduction due to mutations in *HCN4*, while shorter cycle lengths due to adrenergic stimulation may partially counteract an increase in *I*_f_. Furthermore, a prolongation of diastolic depolarization will also be counteracted by a continuing activation of *I*_Ca,L_ and deactivation of *I*_Kr_ (Figure 2D). Overall, the effects of mutations in *HCN4* and adrenergic stimulation are likely exaggerated by the use of a single fixed action potential waveform.

Second, our mathematical model of human SAN *I*_f_ [38,39] is inevitably based on a highly limited amount of experimental data. These data were obtained from a small number of SAN pacemaker cells that were isolated from a single patient with inappropriate tachycardias originating from the SAN region [37]. As such, there may have been abnormalities in the electrophysiology of these cells. Although the sudden onset and termination suggested that the tachycardias were based on reentrant excitation, it cannot be excluded that these tachycardias have resulted in *I*_f_ remodeling of the SAN pacemaker cells [4].

Third, the *in vitro* data on the effects of mutations in *HCN4* are often incomplete. For example, data on the *I*_f_ reversal potential are mostly not provided. The same holds for data on the activation or deactivation rate and the effects of adrenergic stimulation (*cf*. Table 2). In such a case, one can only assume that these are not affected by the mutation of interest.

Despite these limitations, we preferred to study *I*_f_ in simulated action potential clamp experiments, thus ensuring that the action potential followed the course of that of a human SAN pacemaker cell. An alternative would be to incorporate the human *I*_f_ equations into one of the available comprehensive mathematical models of a SAN cell, which are mostly rabbit SAN cells, but one should realize that the thus obtained data on the effects of *I*_f_ on cycle length are largely dependent on the “model environment” [43].

## 4. Some Concluding Remarks

Since the publication of our previous review [30], a large number of novel *HCN4* mutations and variants have been found. The new findings have extended our view. In particular, it has become clear that *HCN4* mutations are accompanied by more cardiac abnormalities than just sinus bradycardia and AF. Newly-found abnormalities include ventricular non-compaction [27,28], prolapse of the mitral valve [27,28] and AV block [25]. Yet, the general picture of *HCN4* mutations as heterozygous, dominant-negative or dominant-negative-like, loss-of-function mutations remains.

Although our insights are far from complete, there are experimental data that not only relate functional loss of HCN4 to sinus bradycardia, but also to AF and AV block. For example, tachycardia-induced remodeling of ion channel expression may lead to down-regulation of *I*_f_ in dogs [44]. On the other hand, chronic AF causes an up-regulation of *I*_f_ in humans [45]. Furthermore, functional loss of HCN4, which is abundantly expressed in the human AV node [46], has been related to AV block in an inducible cardiac-specific HCN4 knockout mouse model [47].

The ventricular non-compaction and mitral valve prolapse point to a role of *HCN4* in the development of the myocardium and a role for dysfunctional HCN4 in structural abnormalities. Indeed, HCN4 is highly expressed throughout the human ventricle at early embryonic stages [48]. However, it is as yet unsolved through which mechanism(s) mutations in *HCN4* lead to LVNC, which is a genetically heterogeneous disorder [49] and may also involve the right ventricle [27,28].

The structural determinants of HCN channel function are complex, and numerous key questions about HCN channel function are still unanswered (for detailed reviews, see Biel *et al.* [50] and He *et al.* [8]). The increasing number of mutations in *HCN4* identified in patients indicates that the pore-forming loop is a hotspot for mutations with severe functional effects, mostly negative shifts in activation and reduced channel expression. The exact molecular mechanisms of the functional defects are as yet unsolved, but the highly conserved GYG motif at Positions 480–482 of the HCN4 protein appears a major determinant of proper channel function. As expected, HCN4 mutations resulting in a lacking or truncated cNBD, *i.e.*, 573X and 695X, respectively, lack cAMP sensitivity [2,22]. Accordingly, the contributions of the 573X and 695X mutant *I*_f_ were not increased upon adrenergic stimulation (Figure 4B).

Mutations or variants in the *N*-terminus or in the distal end of the *C*-terminus seem to have less severe functional effects. This holds in particular for the ten variants identified by Macri *et al.* [26]. Nine did not affect the HCN4 channel characteristics as assessed in patch-clamp experiments (Table 2) and, thus, appeared benign. The kinetic properties were also not affected by the tenth variant, *i.e.*, P257S, but a trafficking defect gave rise to a reduced expression of HCN4 at the membrane. Interestingly, the residue at Position 257 is located in the caveolin-binding domain, and a trafficking defect is in line with the effects that were previously reported for artificially generated mutations in that domain [51]. Thus, the P257S variant does not have a dominant-negative effect on channel function, and its dysrhythmic mechanism is limited to haploinsufficiency. Of note, the HCN4 current was not reduced in patch-clamp experiments on heteromerically expressed P257S channels, which may point to limitations in the use of the CHO cell expression system.

With an increasing amount of data, inconsistencies remain. These include previously identified inconsistencies between clinical and experimental data [30], as well as novel inconsistencies that arise from recent data. Inconsistencies between clinical and experimental data may arise from a relatively low expression or even a complete lack of HCN4 or *I*_f_ channel modulatory elements in the expression systems that are commonly used to assess mutation effects. These elements include MiRP1, PIP_2_, caveolin-3 and SAP-97 [52,53,54,55,56]. Inconsistencies may also arise because experimental data are often incomplete (*cf*. Table 2). In future studies involving HCN4 mutations, it may be important to follow a standardized approach, always including data on trafficking, cAMP sensitivity and kinetic properties, using appropriate experimental protocols. Apparent inconsistencies may also arise, because clinical data are, in most cases, limited to a small number of patients or even a single index patient (*cf*. Table 1). Furthermore, HCN4, although abundant, is not the sole member of the HCN1–HCN4 family in the human SAN [11]. It is therefore conceivable that a considerable amount of HCN tetramers is not fully built from HCN4 subunits, which may not only be important for the behavior of the wild-type current [57], but also for the mutant current.

Inconsistencies arising from recent experimental data are perhaps most striking in patch-clamp data on the G482R mutation in *HCN4* obtained by Milano *et al.* [27] and Schweizer *et al.* [28], who studied heteromerically expressed G482R channels in CHO and HEK-293 cells, respectively. While Milano *et al.* [27] found a strong hyperpolarizing shift in voltage dependence, Schweizer *et al.* [28] found a strong reduction in fully-activated current without such a shift. As emphasized by DiFrancesco [7], differences in protocols to measure activation curves can provide a likely explanation for differences in experimental findings from patch clamp experiments. Furthermore, differences in expression systems, recording temperatures, bath and pipette solutions and patch clamp techniques (whole-cell *vs.* perforated patch) can also contribute to the observed differences. Although an explanation for the differences in patch-clamp data between the Milano *et al.* [27] and Schweizer *et al.* [28] studies remains speculative, the essential difference in observed HCN4 current properties translates into marked differences in the reconstructed *I*_f_ traces (Figure 3F,G), as well as the charge carried by *I*_f_ (Figure 4).

In conclusion, the ongoing identification of *HCN4* mutations in relation to cardiac abnormalities has not only provided us with valuable information, but also with intriguing new questions regarding the role of *HCN4* and *I*_f_ in the human heart.
